# Is drop-out from obesity treatment a predictable and preventable event?

**DOI:** 10.1186/1475-2891-13-13

**Published:** 2014-02-03

**Authors:** Ottavia Colombo, Virginia Valeria Ferretti, Cinzia Ferraris, Claudia Trentani, Piergiuseppe Vinai, Simona Villani, Anna Tagliabue

**Affiliations:** 1 Human Nutrition and Eating Disorder Research Center, Department of Public Health, Experimental and Forensics Medicine, University of Pavia, via A. Bassi, 21, I-27100 Pavia, Italy; 2 Biostatistics and Epidemiology Unit, Department of Public Health, Experimental and Forensics Medicine, University of Pavia, via Forlanini, 2, I-27100 Pavia, Italy; 3 Studi Cognitivi" Post Graduate Cognitive Psychotherapy School Research Group, Milan, Italy; 4 “GNOSIS” Research and Psychotherapy Group V, Mondovì, Italy

**Keywords:** Obesity treatment, Drop-out, Attrition rate, Predictors, Early weight loss

## Abstract

**Background:**

Attrition is an important but understudied issue that plays a vital role in the successful treatment of obesity. To date, most studies focusing on attrition rates and/or its predictors have been based on pretreatment data routinely collected for other purposes. Our study specifically aims at identifying the predictors of drop-out focusing on empirically or theoretically-based factors.

**Methods:**

We conducted a retrospective observational study in an academic outpatient clinical nutrition service in Pavia, Italy. We examined a total of 98 adult obese patients (36 males, 62 females) who underwent a 6-month dietary behavioral weight-loss treatment at our Center. Pre-treatment and treatment-related variables were collected or calculated from clinical charts in order to discriminate those subjects who completed treatment from those who abandoned it before its completion. Multivariable regression analysis was used to identify the independent predictors of drop-out.

**Results:**

The drop-out rates were 21% at 1 month and 57% at 6 months. Compared with completers, noncompleters were significantly younger in terms of age at first dieting attempt (24.0 ± 10.7 *vs.* 31.3 ± 11.2 years, *P* = 0.005), had lower diastolic blood pressure (87.8 ± 9.7 *vs.* 92.7 ± 11.4 mmHg, *P* = 0.022), had a lower baseline body fat percentage (38.5 ± 6.4 *vs.* 41.2 ± 4.4% weight, *P* = 0.015), and had a lower percentage of early weight loss (-1.8 ± 1.8% *vs.* -3.1 ± 2.1%, *P* = 0.035). Moreover, noncompleters significantly differed from completers with regard to type of referral (34.1% *vs.* 53.3% sent by a physician, *P* = 0.036) and SCL-90 anger-hostility subscale (0.83 ± 0.72 vs. 0.53 ± 0.51, *P* = 0.022). A multivariable logistic regression analysis including pre-treatment variables showed that body fat percentage (*P* = 0.030) and SCL-90 anger-hostility subscale (*P* = 0.021) were independently associated with attrition. In a multivariable model considering both pre-treatment and treatment-related factors, attrition was found to be independently related to the age at first dieting attempt (*P* = 0.016) and the achievement of early weight loss (*P* = 0.029).

**Conclusions:**

Our data confirm that psychopathological tracts, early dieting attempts, and a poor initial treatment response are key independent predictors of drop-out from obesity treatment.

## Introduction

The treatment of obesity continues to present major challenges, including poor adherence to diet, inadequate and unsatisfactory weight loss, weight regain, and high rates of attrition. The literature on attrition in the treatment of obesity is heterogeneous, with ranges varying from 10% to 80% depending on the setting and the type of program [1]. Notably, intervention trials reported a mean attrition rate of more than 40% within the first 12 months [2].

To date, most studies focusing on attrition rates and/or its predictors have been based on pre-treatment data (e.g., weight and presence of comorbidities) routinely collected for other purposes (e.g., identifying predictors and correlates of weight loss) [3-8]. High drop-out rates from obesity treatment have been associated with baseline characteristics such as young age [3,9-11], low education levels [6,9,12], poor dieting behaviors [3,5,8,12,13], and unhealthy lifestyles [12-15]. However, the literature on predictors of attrition in obesity treatment is limited by different study designs and inconsistencies across studies.

Emerging evidence suggests that psychological distress (including depression, anxiety, and low self-esteem) [4,7,9,12,16-19] and the failure to achieve reasonable weight loss goals during the first weeks of treatment [4-6,9,12,13,15] are important predictors of drop-out. Previous studies have also reported high rates of psychopathology both in obese [20-22] and normal-weight persons [23] wishing to lose weight. These findings suggest that factors related to the psychological well-being need to be carefully considered so that obese patients keep attending their weight loss program.

The present study specifically aims at identifying the predictors of drop-out from obesity treatment focusing on empirically (e.g., unsatisfactory early weight loss) or theoretically-based (e.g., psychopathological symptoms and dieting behaviors) factors. Because some results may be population-specific [24,25], we also sought to replicate in an Italian academic setting the previously identified predictors of drop-out [1,3-19].

## Methods

### Study design and sample

This research was designed as a retrospective observational study. Participants were adult (age ≥ 18 years) obese patients (body mass index [BMI] ≥ 30 kg/m^2^) who were consecutively referred for weight loss to the Human Nutrition Research Centre, University of Pavia (Italy) between January 2006 and December 2008. Exclusion criteria were pregnancy, major physical illnesses, and psychiatric disorders requiring treatment with atypical antipsychotics. All of the participants underwent a standardized psychological and nutritional assessment before starting a dietary/behavioral weight loss protocol. This study was conducted according to the tenets of Helsinki Declaration and all procedures were approved by the Institutional Review Board of the University of Pavia. Written informed consent was obtained from all participants.

### Weight loss protocol

The weight loss protocol consisted in a 6-month dietary behavioral program. A dietary plan was prepared by a registered dietician for every patient at the beginning of the program; the dietary plan consisted of an individualized balanced low-calorie diet. The energy content was calculated to obtain a weight loss of 0.5 to 1 kg/wk within 6 months according to the Clinical guidelines for the treatment of obesity [26]. The macronutrient composition targets were 25-30% for fats, 55-65% for carbohydrates and 15% for proteins according to the Dietary Reference Values [27]. The diets were calculated using Dieta Ragionata 7.0 (Esi Stampa Medica srl, San Donato Milanese, Italy) which includes the Italian food composition tables. The patients were required to visit our center on a monthly basis for follow-up visits, which were conducted by a physician specialized in clinical nutrition. During the visits, we checked patient body weight and body composition and, when necessary, delt with obstacles hindering weight loss and physical activity. Moreover, possible changes to the dietary plan were discussed with the dietician.

### Predictors of drop-out

The potential predictors of drop-out were collected and/or calculated from clinical charts. Both pre-treatment (i.e., socio-demographic, nutritional, psychopathological features) and treatment-related variables (i.e., achievement of early weight loss at one month) were considered for the purpose of analysis. The following data were collected: socio-demographic characteristics (age, sex, marital status, education level, occupational status, smoking habits, physical activity), anthropometric and nutritional variables (weight, height, BMI, waist and hip circumference, waist-to-hip ratio [WHR]), baseline body composition (percentage body fat [%BF] from skinfold thickness), lower and higher weight achieved during adulthood, age at first dieting attempt, number and reasons of previous attempts to lose weight, type of referral, weight loss goals, and components of the metabolic syndrome. The psychopathological features were assessed by means of validated psychometric instruments. In all participants, we assessed weight loss goals and the achievement of early weight loss at 1 month.

### Psychometric tests

The Symptoms Checklist-90-R (SCL-90-R), a self-report checklist inquiring about symptoms during the preceding week, was used as a measure of general psychopathology [28]. Depressive symptoms and eating behaviors were determined using the Beck Depression Inventory (BDI) [29] and the Binge Eating Scale (BES) [30], respectively.

### Statistical analysis

Categorical variables were expressed as percentages and compared using the chi-squared test or the Fisher’s exact test, as appropriate. Continuous data were summarized as means and standard deviations. Unpaired Student’s *t*-tests (with Satterthwaite’s correction for degrees of freedom) or the Mann-Whitney’s *U* tests were applied to assess the differences in quantitative variables between patients who dropped out at 1 and 6 months and treatment completers. The net changes in the study variables at 6 months were expressed as percentages of the baseline data and compared using paired Student’s *t*-tests. We used multivariable logistic regression analysis to identify the independent predictors of attrition. All of the variables which were significantly associated with attrition (*P* < 0.05) in univariate analyses were selected into the multivariable model. All calculations were performed using the statistical softwares Stata 10 (StataCorp LP, College Station, TX, USA) and SPSS 14.0 (SPSS Inc., Chicago, IL, USA). A two-tailed *P* value < 0.05 was considered statistically significant.

## Results

### General characteristics of completers and noncompleters

Ninety-eight obese adults (36 males and 62 females) were included in the study. The total drop-out rate at one month was 21 (21.4%), without significant sex differences (27.8% in males and 17.7% in females; χ^2^ = 1.4, *P* = 0.243). At 6 months, the total drop-out rate (including subjects who dropped-out at 1 month) was 57% (56 subjects). Again, we found no evidence of a significant sex difference (55.6% in males and 58.1% in females; χ^2^ = 0.1, *P* = 0.809). Table 1 shows the general characteristics of the entire study cohort, divided in completers and noncompleters. Subjects who did not complete the weight loss program were significantly younger at their first dieting attempt; moreover, they had lower%BF (skinfold thickness) and diastolic blood pressure values compared with completers. No other significant intergroup differences were noted in terms of clinical, nutritional, socio-demographic, and lifestyle-related characteristics.

**Table 1:** Baseline general and nutritional characteristics of the entire study cohort stratified according to the presence or absence of attrition (noncompleters and completers)

	**Entire cohort (n = 98)**	**Noncompleters (n = 56)**	**Completers (n = 42)**	***t*-test and *P *** **value**
Age (years)	44.8 ± 14.2	43.1 ± 15.0	47.2 ± 12.7	t = 1.5, p = 0.150
Weight (Kg)	98.3 ± 17.5	97.3 ± 14.9	99.7 ± 20.6	t = 0.6, p = 0.530
Height (cm)	166.8 ± 9.7	166.9 ± 9.0	166.6 ± 10.6	t = -0.2, p = 0.879
Body mass index (kg/m^2^)	35.2 ± 4.8	34.9 ± 4.5	35.7 ± 5.1	t = 0.8, p = 0.413
Systolic blood pressure (mmHg)	136.5 ± 14.9	134.1 ± 14.5	139.8 ± 15.1	t = 1.9, p = 0.064
Diastolic blood pressure (mmHg)	89.9 ± 10.7	87.8 ± 9.7	92.7 ± 11.4	t = 2.3, p = 0.022
Waist circumference (cm)	110.1 ± 12.5	109.1 ± 11.9	111.4 ± 13.3	t = 0.9, p = 0.382
Hip circumference (cm)	119.5 ± 12.4	118.7 ± 9.2	120.8 ± 16.4	t = 0.6, p = 0.529
Waist-to-hip ratio (WHR)	0.92 ± 0.10	0.91 ± 0.10	0.93 ± 0.10	t = 0.9, p = 0.384
%BF skinfold thickness	39.6 ± 5.7	38.5 ± 6.4	41.2 ± 4.4	t = 2.5, p = 0.015
Highest weight in adult age (Kg)	100.2 ± 19.7	99.1 ± 16.7	101.6 ± 23.2	t = 0.5, p = 0.592
Lowest weight in adult age (Kg)	66.7 ± 13.1	67.7 ± 13.0	65.5 ± 13.4	t = -0.7, p = 0.475
Age at first dieting attempt (years)	27.4 ± 11.5	24.0 ± 10.7	31.3 ± 11.2	t = 2.9, p = 0.005
Weight loss goal (kg)	23.6 ± 13.2	23.3 ± 11.7	23.9 ± 15.2	t = 0.2, p = 0.838
Weight loss goal (%)	23.1 ± 9.8	23.6 ± 9.3	22.4 ± 10.6	t = -0.5, p = 0.595

Data are expressed as means ± standard deviation and compared using unpaired Student’s *t*-tests.

Of the 42 subjects who completed treatment, more than 50% were referred to our center by a physician, 36.7% by relatives or friends, whereas 10% did not receive any referral. In contrast, only 34.1% of noncompleters was referred by a physician, 36.4% was advised by relatives or friends, whereas 30% did not receive any referral (Fisher’s exact test; *P* = 0.036).

### Psychometric characteristics of completers and noncompleters

Table 2 shows the psychometric characteristics of the entire study cohort, divided in completers and noncompleters. Compared with completers, subjects who did not complete the weight loss program had significantly higher scores at the SCL-90 anger-hostility subscale. Although there was a marginally significant difference in BES scores between the two groups, no other significant psychometric differences were found.

**Table 2:** Baseline psychometric characteristics of the entire study cohort stratified according to the presence or absence of attrition (noncompleters and completers)

	**Entire cohort (n = 98)**	**Noncompleters (n = 56)**	**Completers (n = 42)**	***t*-test and *P *** **value**
SCL-90				
*Somatization*	0.87 ± 0.70	0.78 ± 0.64	0.98 ± 0.77	t = 1.32, p = 0.189
*Obsessivity-compulsivity*	0.74 ± 0.58	0.83 ± 0.57	0.63 ± 0.57	t = −1.66, p = 0.101
*Interpersonal sensitivity*	0.71 ± 0.68	0.82 ± 0.71	0.57 ± 0.31	t = −1.73, p = 0.087
*Depression*	0.81 ± 0.69	0.91 ± 0.70	0.68 ± 0.67	t = −1.60, p = 0.112
*Anxiety*	0.71 ± 0.62	0.80 ± 0.66	0.60 ± 0.56	t = −1.51, p = 0.135
*Anger-hostility*	0.70 ± 0.65	0.83 ± 0.72	0.53 ± 0.51	t = −2.33, p = 0.022
*Phobic anxiety*	0.29 ± 0.49	0.29 ± 0.51	0.29 ± 0.46	t = −0.00, p = 0.998
*Paranoid ideation*	0.66 ± 0.60	0.68 ± 0.64	0.64 ± 0.56	t = −0.31, p = 0.755
*Psychoticism*	0.39 ± 0.46	0.43 ± 0.49	0.34 ± 0.44	t = −0.93, p = 0.358
*GSI*	0.67 ± 0.51	0.73 ± 0.52	0.60 ± 0.49	t = −1.20, p = 0.233
BES	9.4 ± 7.0	12.7 ± 9.1	9.2 ± 7.1	t = −1.9, p = 0.059
BDI	11.2 ± 8.5	9.8 ± 7.1	8.9 ± 6.9	t = −0.6, p = 0.531

SCL-90: Symptom Checklist-90; GSI: Global Severity Index; BES: Binge Eating Scale; BDI: Beck Depression Inventory.

### Weight loss

**Figure 1:**
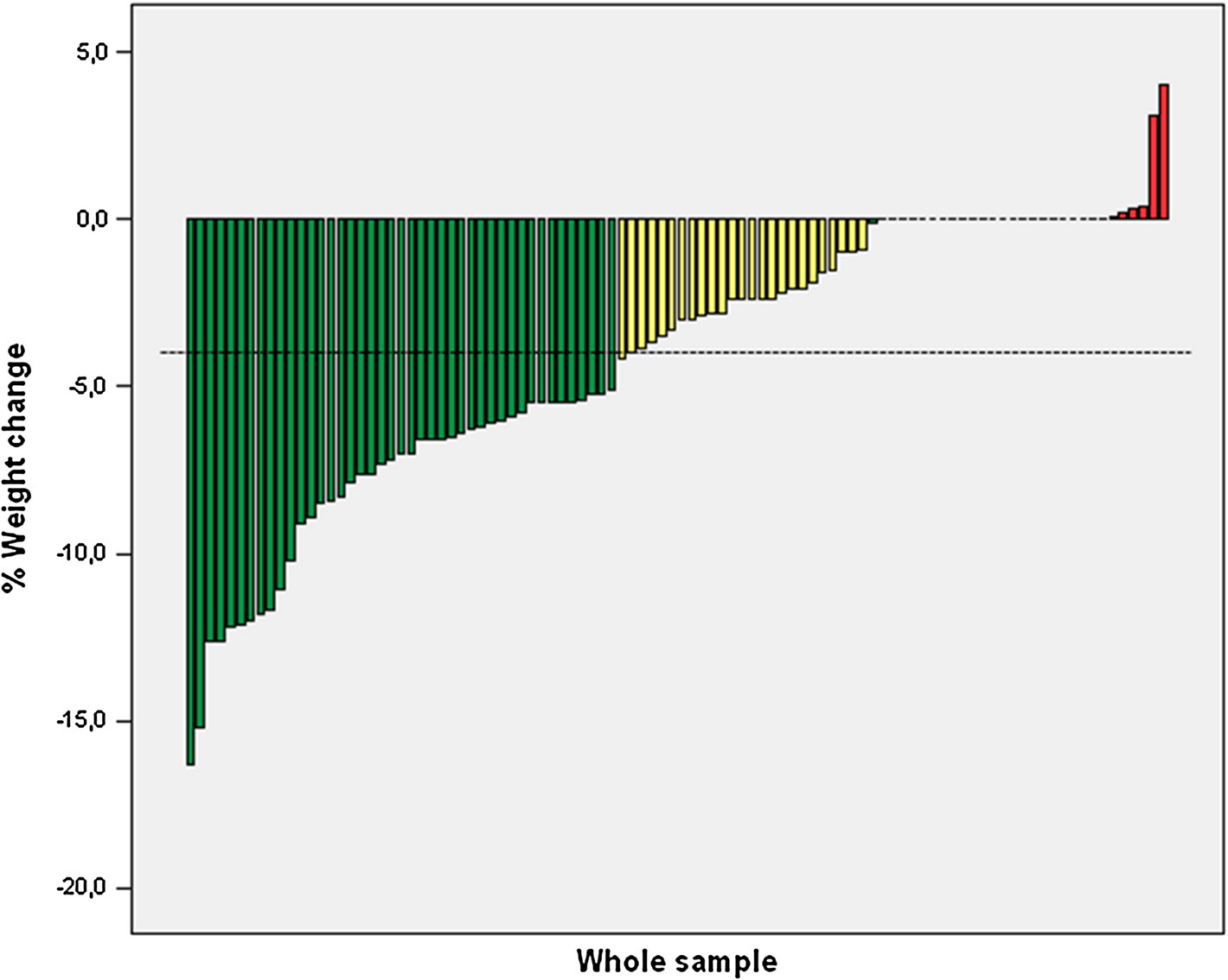
Percentage of weight change in the 98 study participants observed during the entire study period (weight registered at the last visit attended vs. weight at the first visit).

Independent of treatment length, 69 of the study participants (70.4%) showed weight loss, 23 (23.5%) did not show significant weight changes, whereas the remaining 6 subjects (6.1%) had weight gain (Figure 1). The weight loss was greater than 5% of the initial body weight in 43 of the study subjects (43.9%). The mean weight loss percentage was 4.1 ± 4.2% (ranging between +4.0% and -16.3%), whereas the mean early weight loss percentage was 2.5 ± 2.0% (ranging between from +2.0% to -8.8%). Compared with noncompleters, completers showed higher percentages of weight loss both at one month (-3.1 ± 2.1% *vs.* -1.8 ± 1.8%, *P* < 0.01) and at the end of treatment (-7.3 ± 4.1% vs. -1.7 ± 2.4%, *P* < 0.001).

### Predictors of drop-out

A multivariable logistic regression analysis including pre-treatment variables (Table 3) showed that body fat percentage (*P* = 0.030) and the SCL-90 anger-hostility subscale (*P* = 0.021) were independently associated with an increased likelihood of attrition. The risk of drop-out was reduced by approximately 15% for each one percent increase in body fat, whereas there was a 4-fold increase in the risk of drop-out for each 1-point in the SCL-90 anger-hostility subscale. In a multivariable model considering both pre-treatment and treatment-related factors (Table 4), attrition was found to be independently related to the age at first dieting attempt (p = 0.016) and the achievement of early weight loss (p = 0.029). The risk of drop-out decreased by 11% for each 1-year increase in the age at the first dieting attempt. Moreover, the risk of drop-out was reduced by 40% for each 1-point percentage decrease in body weight at one month compared with baseline values.

**Table 3:** Multivariable logistic regression analysis for pre-treatment variables predicting drop-out from obesity treatment

	**Odds ratio**	**95% confidence interval**	***P value***
Age at first dieting attempt	0.95	0.90 − 1.01	0.096
Sex (males *vs*. female)	0.26	0.04 − 1.61	0.149
Diastolic blood pressure	0.98	0.93 − 1.04	0.553
%BF skinfold thickness	0.84	0.72 − 0.98	0.030
SCL-90 anger-hostility subscale	4.61	1.26 − 16.85	0.021

%BF: percentage body fat.

**Table 4:** Multivariable logistic regression analysis for pre-treatment variables and treatment-related factors predicting drop-out from obesity treatment

	**Odds ratio**	**95% confidence interval**	***P value***
Age at first dieting attempt	0.89	0.81–0.98	0.016
Sex (males *vs*. female)	0.17	0.02–1.94	0.155
Diastolic blood pressure	1.00	0.94–1.07	0.980
%BF skinfold thickness	0.81	0.65–1.01	0.059
SCL-90 anger-hostility subscale	3.97	0.79–19.90	0.094
Early weight loss (%)	0.57	0.34–0.95	0.029

%BF: percentage body fat.

## Discussion

The identification and validation of reliable predictors of attrition would be helpful in reducing the drop-out from obesity treatment. In this study, we specifically aimed at identifying the predictors of drop-out focusing on theoretically- (e.g., psychopathological symptoms and dieting behaviors) or empirically-based (e.g., unsatisfactory early weight loss) factors.

Despite the higher drop-out rate (57% at 6 months) observed in our study, our findings are generally in line with those reported in recent studies with a similar treatment duration [5,17,31]. Our results indicate that few baseline variables (%BF, diastolic blood pressure, type of referral, and age at first dieting attempt) significantly differed between completers and noncompleters. It is conceivable that subjects with a higher awareness of their impaired health status may have a lower drop-out rate. Notably, obese subjects are characterized by a high prevalence of psychopathology [23] which may in turn have a significant impact on attrition. Interestingly, our results indicate that higher anger-hostility scores on the SCL-90 are associated with increased drop-out rates, suggesting that anger and anger expression styles may predict attrition among adult obese individuals wishing to lose weight. Different mechanisms may explain the relation between anger-hostility and an increased risk of drop-out. Burns and coworkers [32] reported that hostile patients are characterized by a negative set of expectations and tend to be suspicious of others [33]. Such a suspicious relational style may predispose them to reject the physician’s suggestions [34]. Negative expectations may also undermine the confidence with health care providers and lead to an inability to follow the clinician’s recommendations. Moreover, physicians may be alienated [32] by cynicism and hostility, which can in turn result in poor treatment engagement. Finally, hostility could also contribute to the likelihood of drop-out through its association with negative affect [35].

The high attrition rate (21.4%) observed in our study at one month highlights the paramount role played by baseline motivational status for predicting treatment discontinuation [31,36]. In accordance with previous data [6], we found that the achievement of early weight loss may represent a strong motivational incentive for obtaining clinically meaningful weight loss goals. Taken together, these data confirm that the attrition rates in weight loss programs are not only related to psychopathological variables (i.e., anger) and age at first dieting attempt, but also to treatment-related factors such as the percentage of initial body weight lost after the first weeks of treatment [9]. It is likely that anger and hostility can act as upstream factors of early weight loss, i.e. individuals who show signs of anger will be less likely to comply with the weight loss regimen and therefore lose less weight early in the program. Additionally, both a lower stress threshold and a poorer capability to accept worse-than-expected outcomes (i.e., weight loss) could make those subjects abandon treatment before its completion. Notably, we found a borderline higher BES score in noncompleters, which may suggest a reduced self-control level towards food.

Several previous studies conducted in different settings have been designed to identify the potential baseline predictors of drop-out from obesity treatment. The findings from our current study are generally in agreement with the majority of previous reports in the field [3,7-15,17,18] although the results were not always consistent [6,8,9,12] and sporadically even were at variance [5,11]. There are several potential reasons that may account for the discrepant findings across studies, including differences in the study settings, the number and characteristics of the subjects recruited, and the type of weight loss protocols.

Our report has some limitations that merit consideration. First, this study was based on a convenience sample, which limits the generalizability of its findings. Second, we did not specifically investigate the reasons of drop-out. Finally, our study was conducted on a limited number of Italian obese patients who were enrolled in an obesity treatment program in an outpatient academic setting; therefore, our results might not apply to different settings and need further validation before firm conclusions can be made.

## Conclusion

In summary, the results from the current study indicate that baseline body fat percentage, SCL-90 anger-hostility subscale, and age at first attempt to diet were independent predictors of drop-out from treatment in an Italian cohort of adult obese subjects. Importantly, we also demonstrate that the achievement of early weight loss was independently associated with a reduced attrition rate. These results highlight the importance of a close clinical monitoring in the first weeks of treatment to reduce the attrition rates and make drop-out from obesity treatment not only predictable but also preventable. Future studies are needed to determine whether targeted psychological interventions may reduce attrition rates in obese subjects with significant anger and anger expression styles.

## Abbreviations

BMI: Body mass index; %BF: Percentage body fat; WHR: Waist-to-hip ratio; SCL-90: Symptoms checklist-90; BDI: Beck depression inventory; BES: Binge eating scale.

## Competing interests

The authors declare that they have no competing interests.

## Authors’ contributions

OC participated in the conception and design of the study, analyzed and interpreted the data, and drafted the manuscript; FVV analysed and interpreted the data; CF participated in data collection and interpretation; CT participated in data collection and interpretation; PV participated in data collection and interpretation; SV supervised the design of the study, analyzed and interpreted the data; AT was responsible for the overall scientific direction and supervision. All the authors have read and approved the final manuscript.

## References

[B1] Moroshko I Brennan L O'Brien P Predictors of dropout in weight loss interventions: a systematic review of the literature Obes Rev 2011 12 912 934 10.1111/j.1467-789X.2011.00915.x 21815990

[B2] Dansinger ML Gleason JA Griffith JL Comparison of the Atkins, Ornish, Weight Watchers, and Zone diets for weight loss and heart disease risk reduction; a randomized trial JAMA 2005 293 43 53 10.1001/jama.293.1.43 15632335

[B3] Gunnarsdóttir I Sigurgeirsdóttir GK Thórsdóttir I Predictors of dropping out in a weight loss intervention trial Ann Nutr Metab 2010 56 212 Epublication 11 March 2010 10.1159/000279224 20224274

[B4] Lim SS Norman RJ Clifton PM Psychological effects of prescriptive vs. general lifestyle advice for weight loss in young women J Am Diet Assoc 2009 109 1917 1921 10.1016/j.jada.2009.08.008 19857635

[B5] Messier V Hayek J Karelis AD Anthropometric, metabolic, psychosocial and dietary factors associated with dropout in overweight and obese postmenopausal women engaged in a 6-month weight loss programme: a MONET study Br J Nutr 2010 103 1230 1235 Epublication 24 November 2009 19930768 10.1017/S0007114509993023

[B6] Elfhag K Rossner S Initial weight loss is the best predictor for success in obesity treatment and sociodemographic liabilities increase risk of drop-out Patient Educ Couns 2010 79 361 366 10.1016/j.pec.2010.02.006 20223613

[B7] Chang MW Brown R Nitzke S Participant recruitment and retention in a pilot program to prevent weight gain in low-income overweight and obese mothers BMC Public Health 2009 9 424 10.1186/1471-2458-9-424 19930587 PMC2785793

[B8] Inelmen EM Toffanello ED Enzi G Predictors of drop-out in overweight and obese outpatients Int J Obes 2005 29 122 128 10.1038/sj.ijo.0802846 15545976

[B9] Fabricatore AN Wadden TA Moore RH Predictors of attrition and weight loss success: Results from a randomized controlled trial Behav Res Ther 2009 47 685 691 Epublication 20 May 2009 10.1016/j.brat.2009.05.004 19497559 PMC2713356

[B10] Gripeteg L Karlsson J Torgerson J Predictors of very-low-energy diet outcome in obese women and men Obes Facts 2010 3 159 165 Epublication 11 June 2010 20616605 10.1159/000314655 PMC6452165

[B11] Dalle Grave R Calugi S Molinari E Weight loss expectations in obese patients and treatment attrition: an observational multicenter study Ob Res 2005 13 1961 1969 10.1038/oby.2005.241 16339128

[B12] Bradshaw AJ Horwath CC Katzer L Non-dieting group interventions for overweight and obese women: what predicts non-completion and does completion improve outcomes? Public Health Nutr 2010 13 1622 1628 10.1017/S1368980009992977 20025832

[B13] Greenberg I Stampfer MJ Schwarzfuchs D Adherence and success in long-term weight loss diets: the dietary intervention randomized controlled trial (DIRECT) J Am Coll Nutr 2009 28 159 168 10.1080/07315724.2009.10719767 19828901

[B14] Busetto L Mazza M Salvalaio S Obesity treatment in elderly outpatients: predictors of efficacy and drop-out Eat Weight Disord 2009 14 e56 e65 10.1007/BF03327801 19934638

[B15] Kong W Langlois MF Kamga-Ngandé C Predictors of success to weight-loss intervention program in individuals at high risk for type 2 diabetes Diabetes Res Clin Pract 2010 90 147 153 Epublication 24 July 2010 10.1016/j.diabres.2010.06.031 20655608

[B16] De Panfilis C Cero S Dall’Aglio E Psychopathological predictors of compliance and outcome in weight-loss obesity treatment Acta Biomed 2007 78 22 28 17687813

[B17] De Panfilis C Torre M Cero S Personality and attrition from behavioral weight-loss treatment for obesity Gen Hosp Psychiatry 2008 30 515 520 Epublication 3 August 2008 10.1016/j.genhosppsych.2008.06.003 19061677

[B18] Komulainen T Keränen AM Rasinaho E Quitting a weight loss program is associated with anhedonia: preliminary findings of the Lifestyle Intervention Treatment Evaluation Study in northern Finland Int J Circumpolar Health 2011 70 72 78 Epublication 16 February 2011 21329577 10.3402/ijch.v70i1.17795

[B19] Somerset SM Graham L Markwell K Depression scores predict adherence in a dietary weight loss intervention trial Clin Nutr 2011 30 593 598 Epublication 14 May 2011 10.1016/j.clnu.2011.04.004 21575998

[B20] Marchesini G Bellini M Natale S Psychiatric distress and health-related quality of life in obesity Diabetes Nutr Metab 2003 16 145 154 14635731

[B21] Onyike CU Crum RM Lee HB Is obesity associated with major depression? Results from the Third National Health and Nutrition Examination Survey Am J Epidemiol 2003 158 1139 1147 10.1093/aje/kwg275 14652298

[B22] Scott KM Bruffaerts R Simon GE Obesity and mental disorders in the general population: results from the world mental health surveys Int J Obes 2008 32 192 200 10.1038/sj.ijo.0803701 PMC2736857 17712309

[B23] Martinelli V Colombo O Nichini C High frequency of psychopathology in subjects wishing to lose weight: an observational study in Italian subjects Public Health Nutr 2011 14 373 376 Epublication 11 June 2010 10.1017/S1368980010001576 20537213

[B24] Teixeira PJ Scott BG Houtkooper LB Weight loss readiness in middle-aged women: psychosocial predictors of success for behavioral weight reduction J Behav Med 2002 25 499 523 10.1023/A:1020687832448 12462956

[B25] Teixeira PJ Palmeira AL Branco TL Who will lose weight? A reexamination of predictors of weight loss in women Int J Behav Nutr Phys Act 2004 1 1 12 10.1186/1479-5868-1-1 15287984 PMC511005

[B26] NHLBI Obesity Education Initiative Expert Panel on the Identification, Evaluation, and Treatment of Overweight and Obesity in Adults Clinical guidelines on the identification, evaluation, and treatment of overweight and obesity in adults – the evidence report. National Institutes of Health Obes Res 1998 6 51S 209S 9813653

[B27] Italian Society of Human Nutrition Recommended levels of assumption of energy and nutrients for the Italian population (LARN) 2012 Rome [http://sinu.it/documenti/20121016_LARN_bologna_sintesi_prefinale.pdf]

[B28] Prunas A Sarno I Preti E Psychometric properties of the Italian version of the SCL-90-R: a study on a large community sample Eur Psychiatry 2012 27 591 597 Epublication 21 February 2011 10.1016/j.eurpsy.2010.12.006 21334861

[B29] Beck AT Depression: Causes and treatment 1967 Philadelphia: University of Pennsylvania Press

[B30] Gormally J Black S Daston S The assessment of binge eating severity among obese persons Addict Behav 1982 7 47 55 10.1016/0306-4603(82)90024-7 7080884

[B31] Minniti A Bissoli L Di Francesco V Individual versus group therapy for obesity: comparison of dropout rate and treatment outcome Eat Weight Disord 2007 12 161 167 10.1007/BF03327593 18227637

[B32] Burns JW Higdon LJ Mullen JT Relationships among patient hostility, anger expression, depression, and the working alliance in a work hardening program Ann Behav Med 1999 21 77 82 10.1007/BF02895037 18425658

[B33] Pope MK Smith TW Rhodewalt F Cognitive, behavioral, and affective correlates of the Cook and Medley Hostility Scale J Pers Assess 1990 54 501 514 10.1207/s15327752jpa5403&4_7 2348338

[B34] Smith TW Hostility and health: current status of a psychosomatic hypothesis Health Psychol 1992 11 139 150 1618168 10.1037//0278-6133.11.3.139

[B35] Pugh R An association between hostility and poor adherence to treatment in patients suffering from depression Br J Med Psychol 1983 56 205 208 10.1111/j.2044-8341.1983.tb01548.x 6882699

[B36] Teixeira PJ Silva MN Mata J Motivation, self-determination, and long-term weight control Int J Behav Nutr Phys Act 2012 9 22 10.1186/1479-5868-9-22 22385818 PMC3312817

